# Supplementary material for: Learning from data to design functional materials without inversion symmetry
Source: Nat Commun. 2017 Feb 17;8:14282. doi: 10.1038/ncomms14282 (PMC5321684; doi:10.1038/ncomms14282)
Supplement: Supplementary Information — Supplementary Figures, Supplementary Tables, Supplementary Notes and Supplementary References [file ncomms14282-s1.pdf]

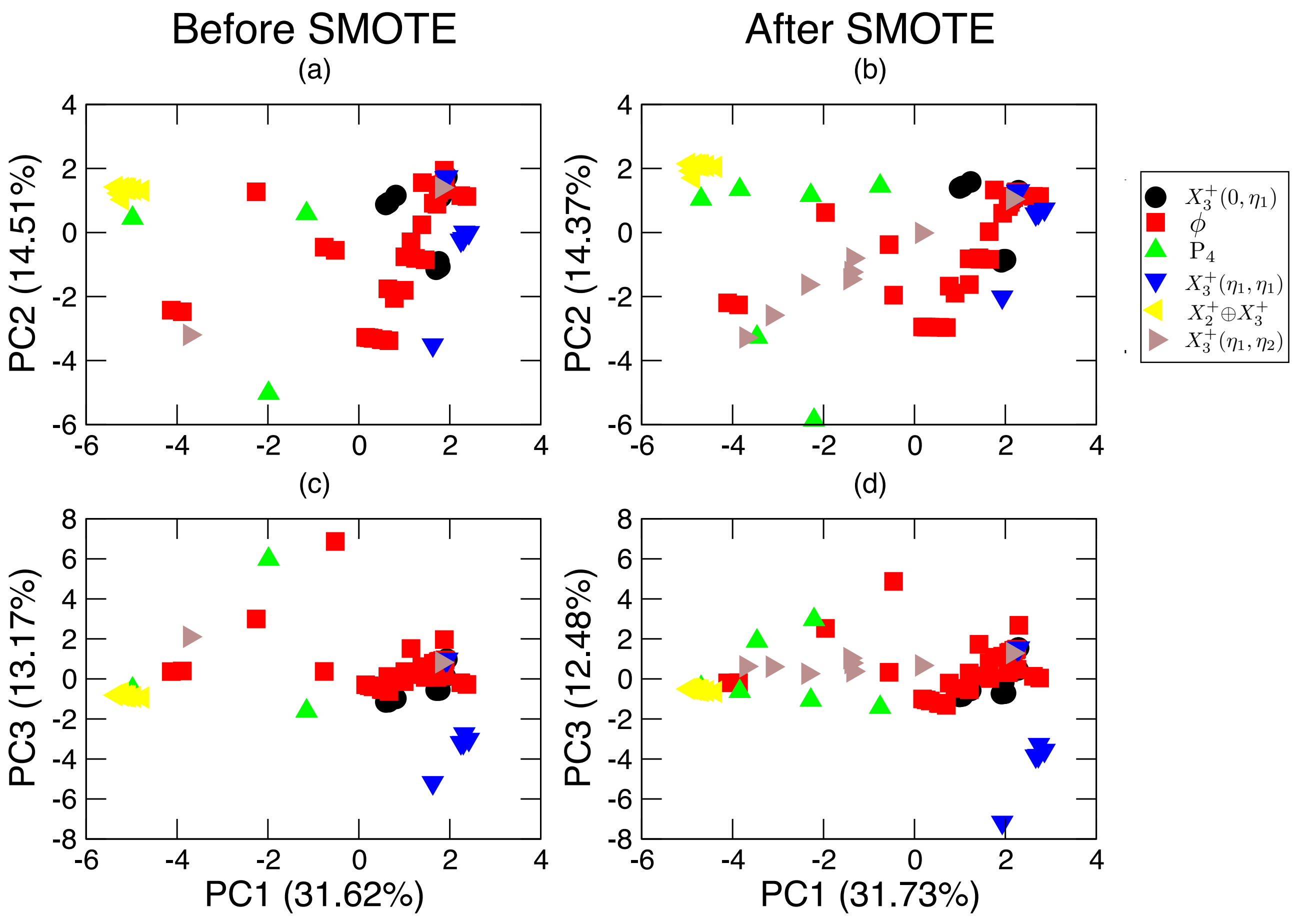

Supplementary Figure 1 | Visualization of our dataset in the principal component analysis (PCA) space before (a) & (c) and after (b) & (d) applying the SMOTE algorithm. Synthetic data points were created for P_4 (green triangle up, △) and X_3^+ (η_1, η_2) (brown triangle right, ▹) irrep labels in (b) and (d). Note that we show -1×PC2 as the ordinate in (b) for the ease of comparison. This simple transformation (rotation by 180°) in PC2 does not affect the outcome of our work. The % variance captured by the top three PC1, PC2 and PC3 before and after SMOTE are 59.3 and 58.58%, respectively, indicating only very little difference between them. Furthermore, key features of the data manifold in (a) and (c) are preserved in (b) and (d). This suggests that SMOTE did not affect the manifold of our original data set.

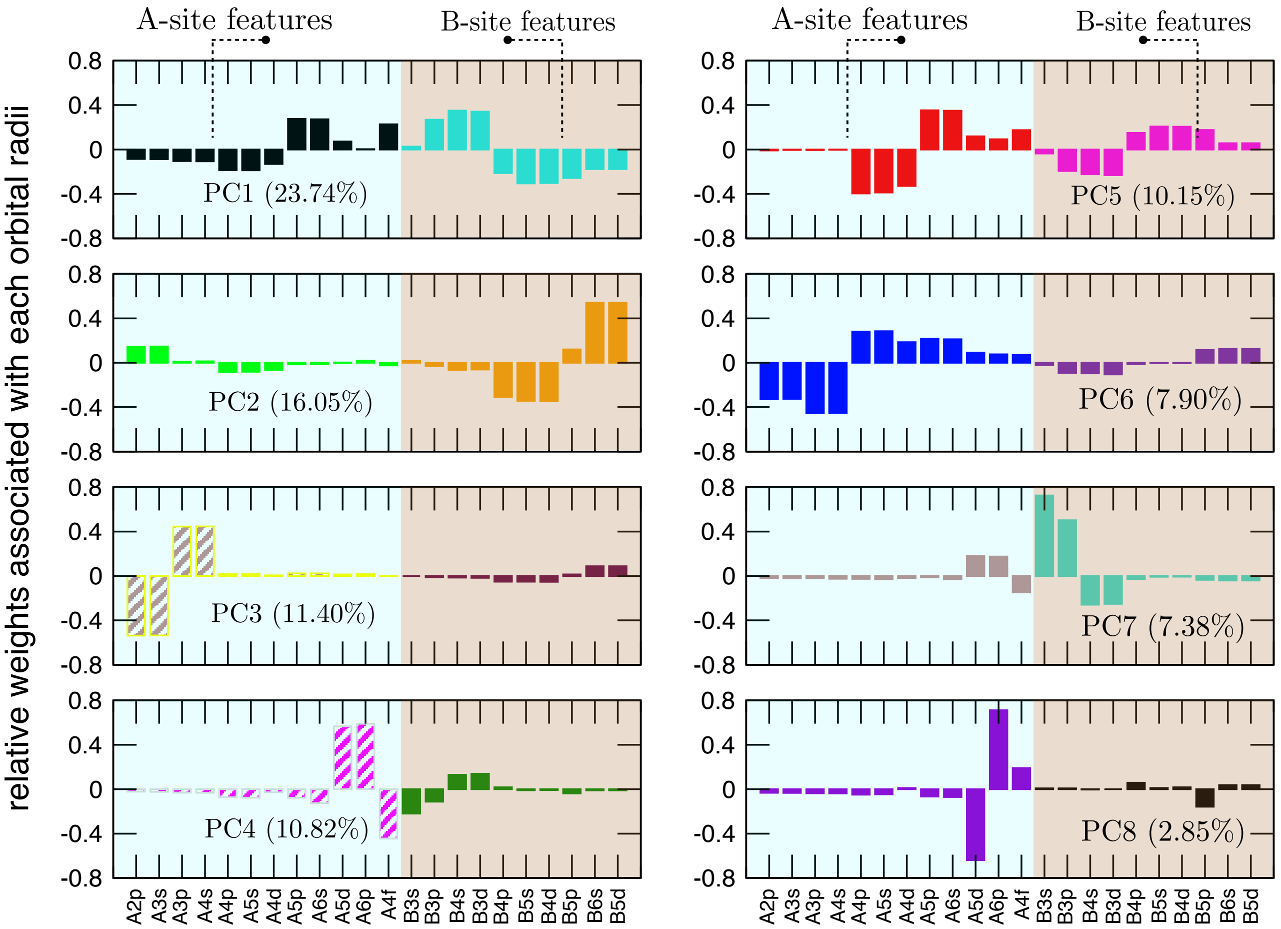

Supplementary Figure 2 | Linear combination of the weighted contributions (coefficients) of orbital radii for the 8 principal components (PCs) along with their variance captured (in %) on a dataset with 3,253 chemical compositions and 22 original orbital radii features. The 8 PCs together capture a total of 91.73% variance in the dataset, thereby significantly reducing the dimensionality of the dataset from 22 to 8. The larger the relative weight (absolute value) of a feature, the greater is its contribution to that PC (e.g. in PC8, radii of $5d$ and $6p$ orbitals for the A-site atoms dominate relative to others).

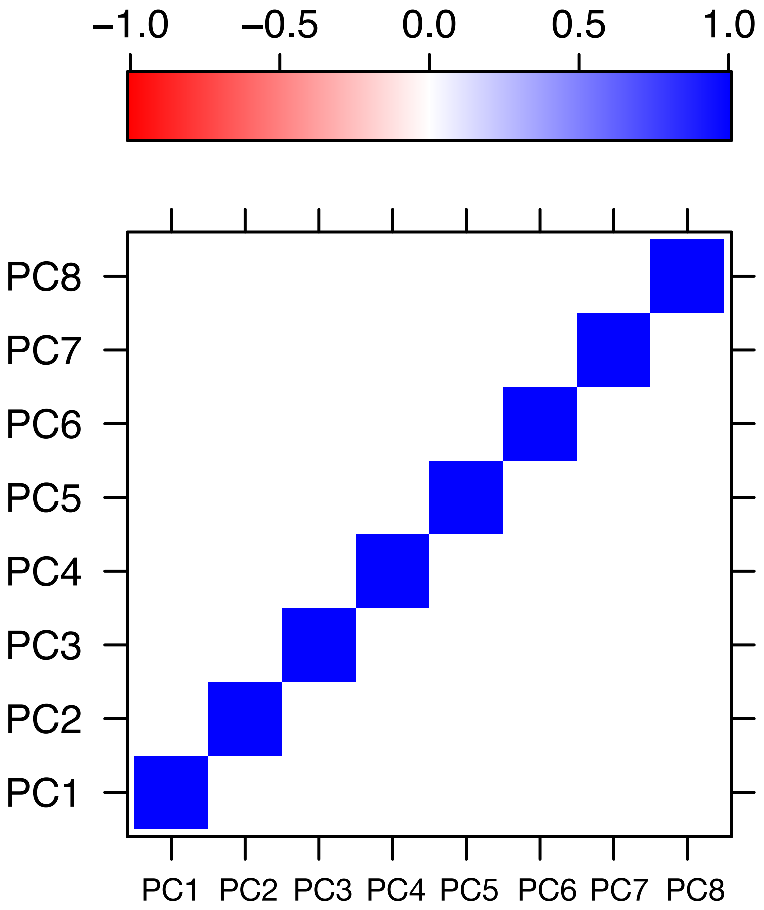

Supplementary Figure 3 | Heat map showing the pairwise statistical correlation between the PCs. Dark blue and red color indicate strong positive and negative correlation, respectively. White color denotes no statistical correlation. The plot shows that there is no statistical correlation between PCs.

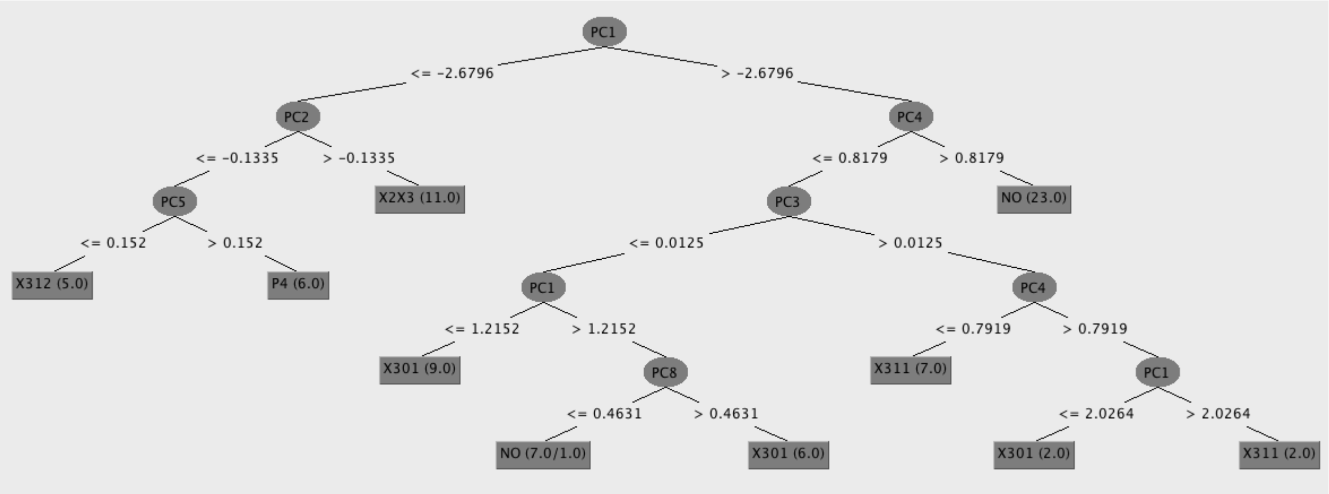

Supplementary Figure 4 | Decision tree 1 from the first bootstrapped sample. The class labels (irreps) are given in the leaf nodes. NCS space groups are obtained via the superposition of $X_2^+ \oplus X_3^+$, X_3^+ (η_1, η_1) and X_3^+ (η_1, η_2) irreps with the M_3^- irrep or A/A′ cation-ordering. See Fig. 3b in the main article for a schematic of the M_3^- A/A′ cation-ordering. On the other hand, CS space groups are obtained even after the superposition of X_3^+ $(0, \eta_1)$, P_4, and ϕ irreps with the M_3^- irrep. The definition of each PC in terms of the linear combination of orbital radii is given in Supplementary Figure 2. The bracketed numbers at each leaf node correspond to the total number of RP compositions that reach the leaf. Sometimes we also find two numbers. The first number is the total number of compositions reaching the leaf node, whereas the second number is the number of misclassified compositions in the same leaf node.

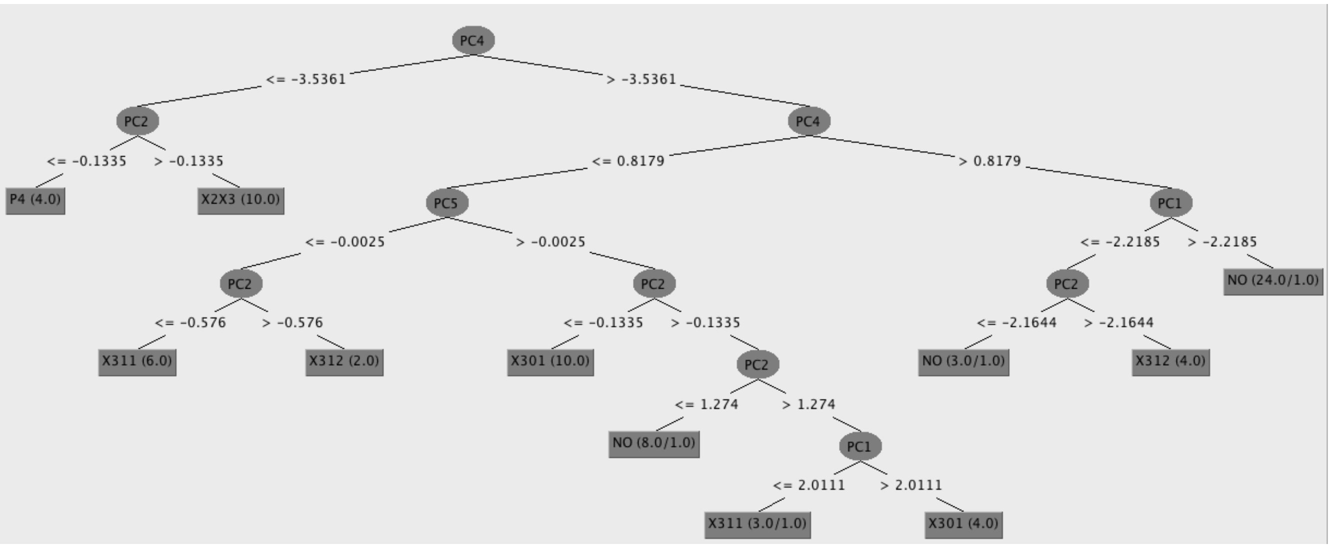

Supplementary Figure 5 | Decision tree 2 from the second bootstrapped sample. The class labels (irreps) are given in the leaf nodes. NCS space groups are obtained via the superposition of $X_2^+ \oplus X_3^+$, X_3^+ (η_1, η_1) and X_3^+ (η_1, η_2) irreps with the M_3^- irrep or A/A′ cation-ordering. See Fig. 3b in the main article for a schematic of the M_3^- A/A′ cation-ordering. On the other hand, CS space groups are obtained even after the superposition of X_3^+ $(0, \eta_1)$, P_4, and ϕ irreps with the M_3^- irrep. The definition of each PC in terms of the linear combination of orbital radii is given in Supplementary Figure 2. The bracketed numbers at each leaf node correspond to the total number of RP compositions that reach the leaf. Sometimes we also find two numbers. The first number is the total number of compositions reaching the leaf node, whereas the second number is the number of misclassified compositions in the same leaf node.

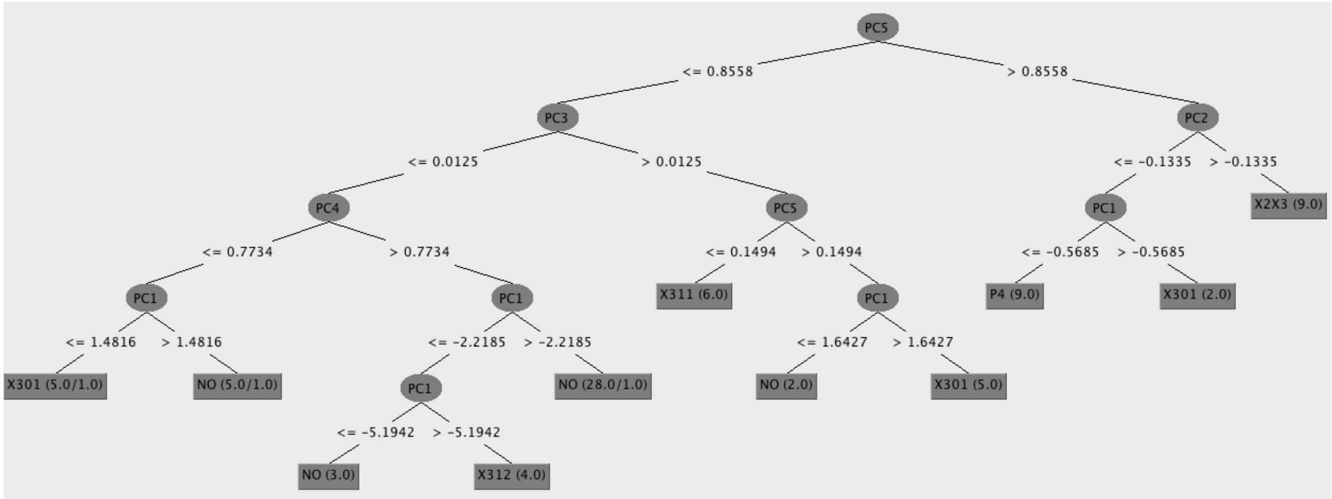

Supplementary Figure 6 | Decision tree 3 from the third bootstrapped sample. The class labels (irreps) are given in the leaf nodes. NCS space groups are obtained via the superposition of $X_2^+ \oplus X_3^+$, X_3^+ (η_1, η_1) and X_3^+ (η_1, η_2) irreps with the M_3^- irrep or A/A′ cation-ordering. See Fig. 3b in the main article for a schematic of the M_3^- A/A′ cation-ordering. On the other hand, CS space groups are obtained even after the superposition of X_3^+ $(0, \eta_1)$, P_4, and ϕ irreps with the M_3^- irrep. The definition of each PC in terms of the linear combination of orbital radii is given in Supplementary Figure 2. The bracketed numbers at each leaf node correspond to the total number of RP compositions that reach the leaf. Sometimes we also find two numbers. The first number is the total number of compositions reaching the leaf node, whereas the second number is the number of misclassified compositions in the same leaf node.

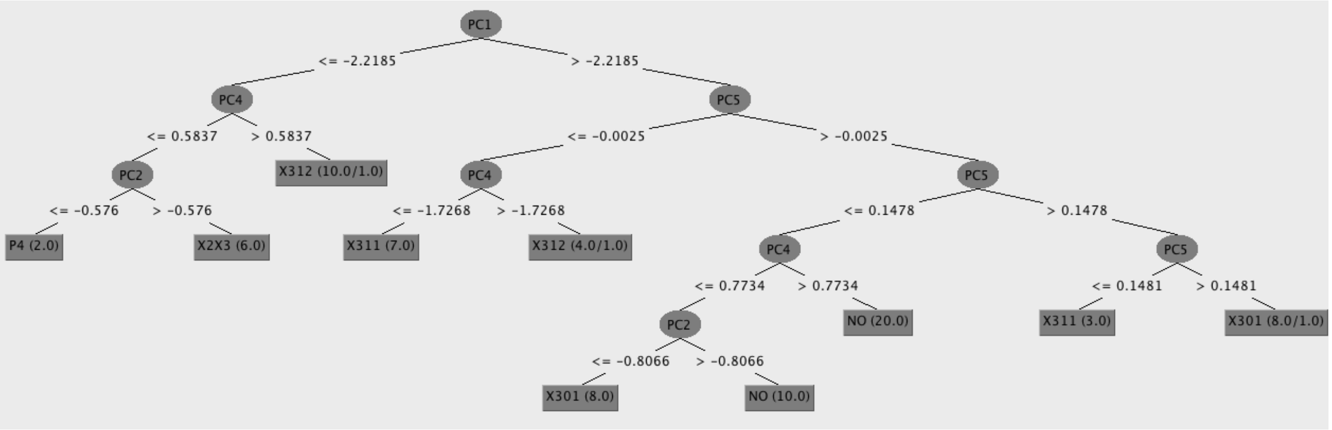

Supplementary Figure 7 | Decision tree 4 from the fourth bootstrapped sample. The class labels (irreps) are given in the leaf nodes. NCS space groups are obtained via the superposition of $X_2^+ \oplus X_3^+$, X_3^+ (η_1, η_1) and X_3^+ (η_1, η_2) irreps with the M_3^- irrep or A/A′ cation-ordering. See Fig. 3b in the main article for a schematic of the M_3^- A/A′ cation-ordering. On the other hand, CS space groups are obtained even after the superposition of X_3^+ $(0, \eta_1)$, P_4, and ϕ irreps with the M_3^- irrep. The definition of each PC in terms of the linear combination of orbital radii is given in Supplementary Figure 2. The bracketed numbers at each leaf node correspond to the total number of RP compositions that reach the leaf. Sometimes we also find two numbers. The first number is the total number of compositions reaching the leaf node, whereas the second number is the number of misclassified compositions in the same leaf node.

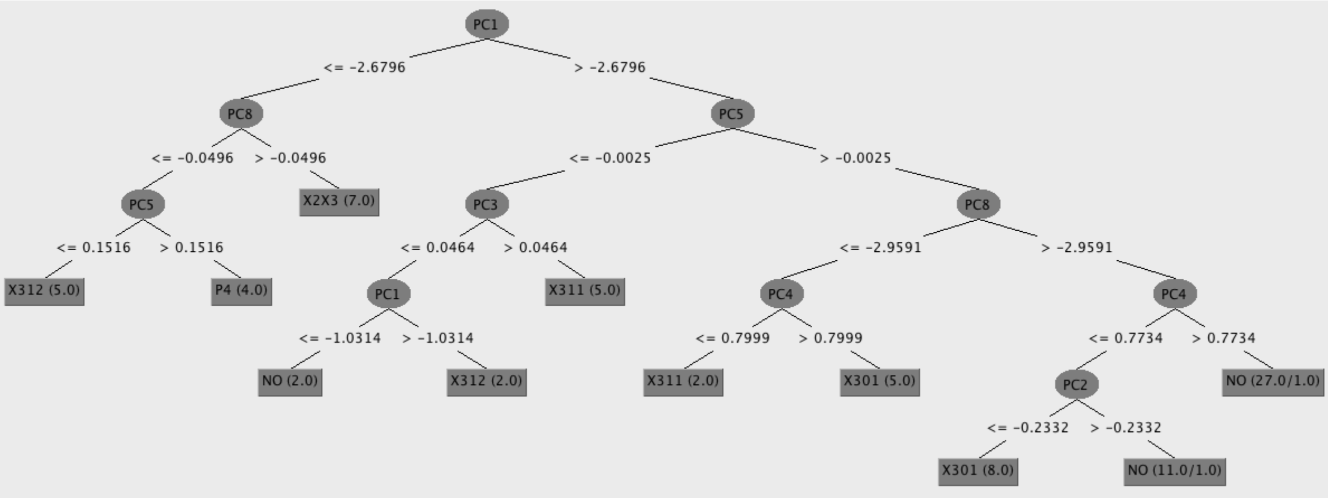

Supplementary Figure 8 | Decision tree 5 from the fifth bootstrapped sample. The class labels (irreps) are given in the leaf nodes. NCS space groups are obtained via the superposition of $X_2^+ \oplus X_3^+$, X_3^+ (η_1, η_1) and X_3^+ (η_1, η_2) irreps with the M_3^- irrep or A/A′ cation-ordering. See Fig. 3b in the main article for a schematic of the M_3^- A/A′ cation-ordering. On the other hand, CS space groups are obtained even after the superposition of X_3^+ $(0, \eta_1)$, P_4, and ϕ irreps with the M_3^- irrep. The definition of each PC in terms of the linear combination of orbital radii is given in Supplementary Figure 2. The bracketed numbers at each leaf node correspond to the total number of RP compositions that reach the leaf. Sometimes we also find two numbers. The first number is the total number of compositions reaching the leaf node, whereas the second number is the number of misclassified compositions in the same leaf node.

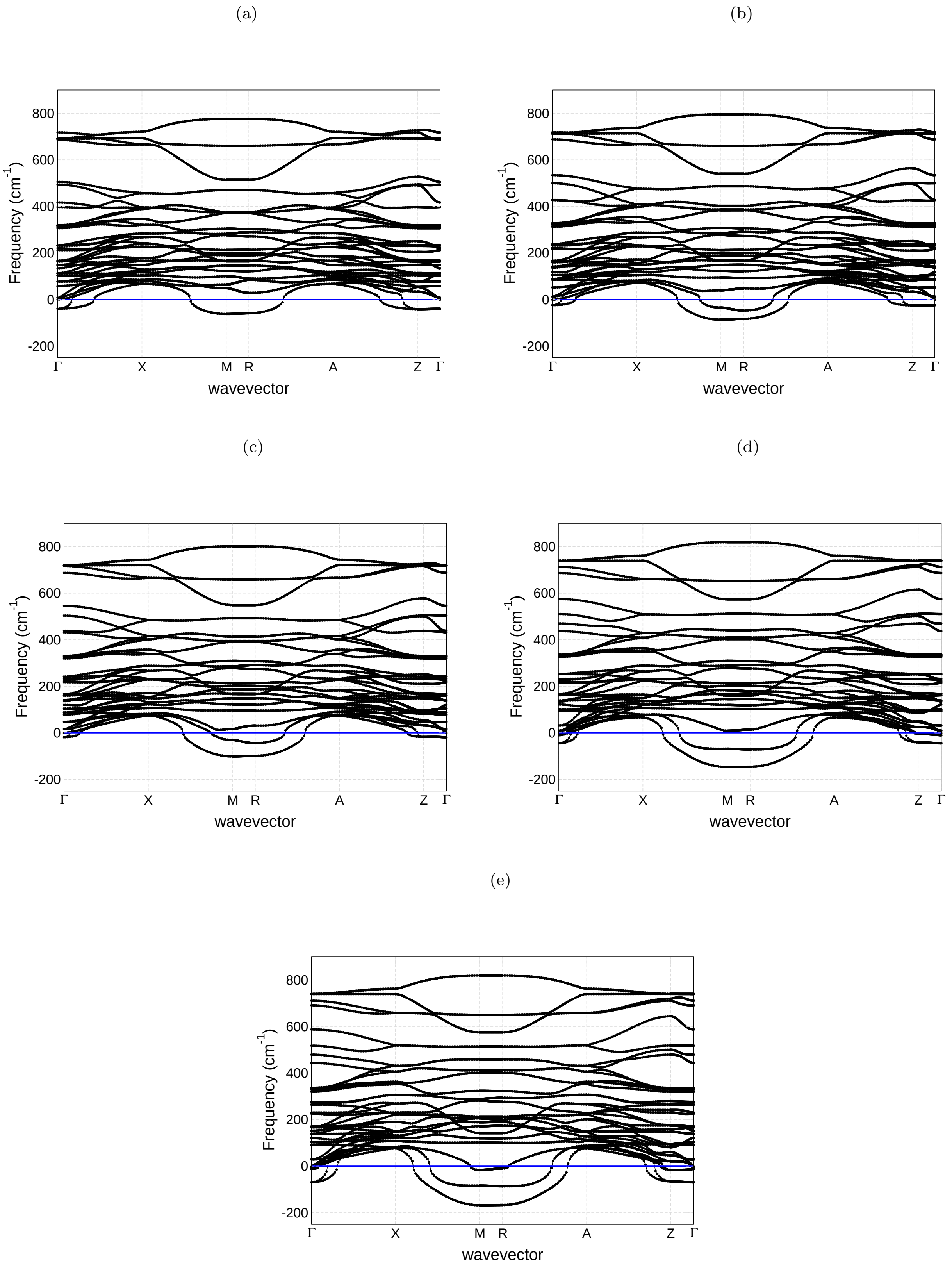

Supplementary Figure 9 | Phonon band structure data for NaRSnO$_4$ in $P4/nmm$ crystal structure, where R=(a) La, (b) Pr, (c) Nd, (d) Gd and (e) Y, using PBEsol exchange-correlation functional.

(a)

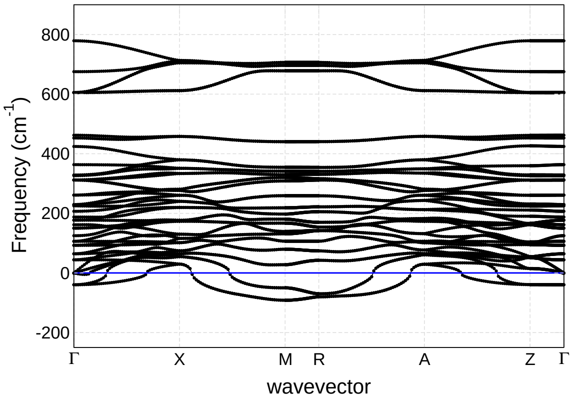

(b)

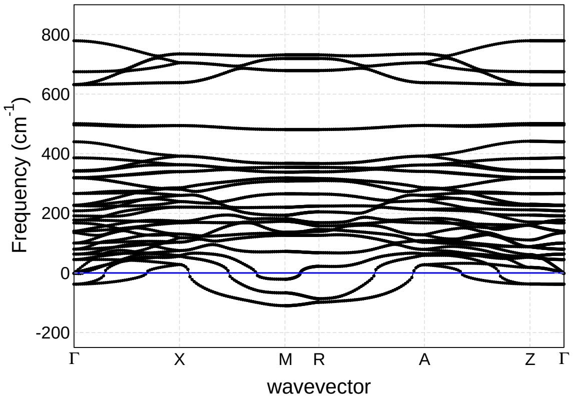

(c)

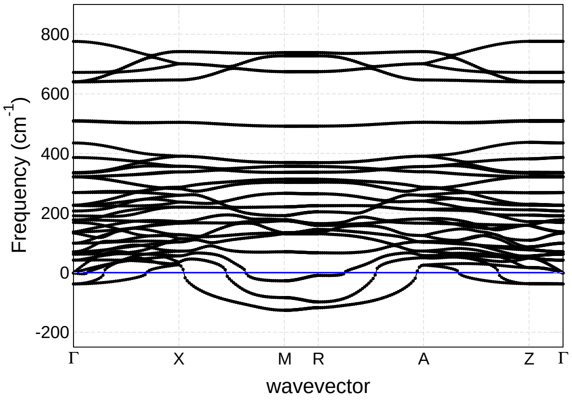

(d)

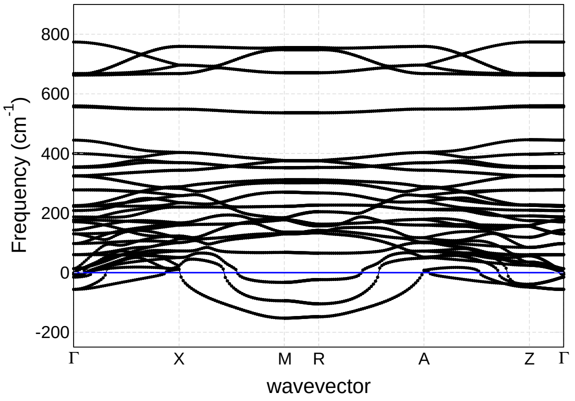

(e)

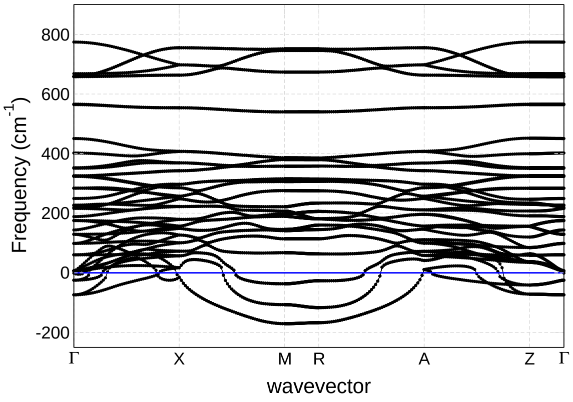

Supplementary Figure 10 | Phonon band structure data for NaRRuO$_4$ in $P4/nmm$ crystal structure, where R=(a) La, (b) Pr, (c) Nd, (d) Gd and (e) Y, using PBEsol exchange-correlation functional. Ferromagnetic spin order was imposed on the Ru atom.

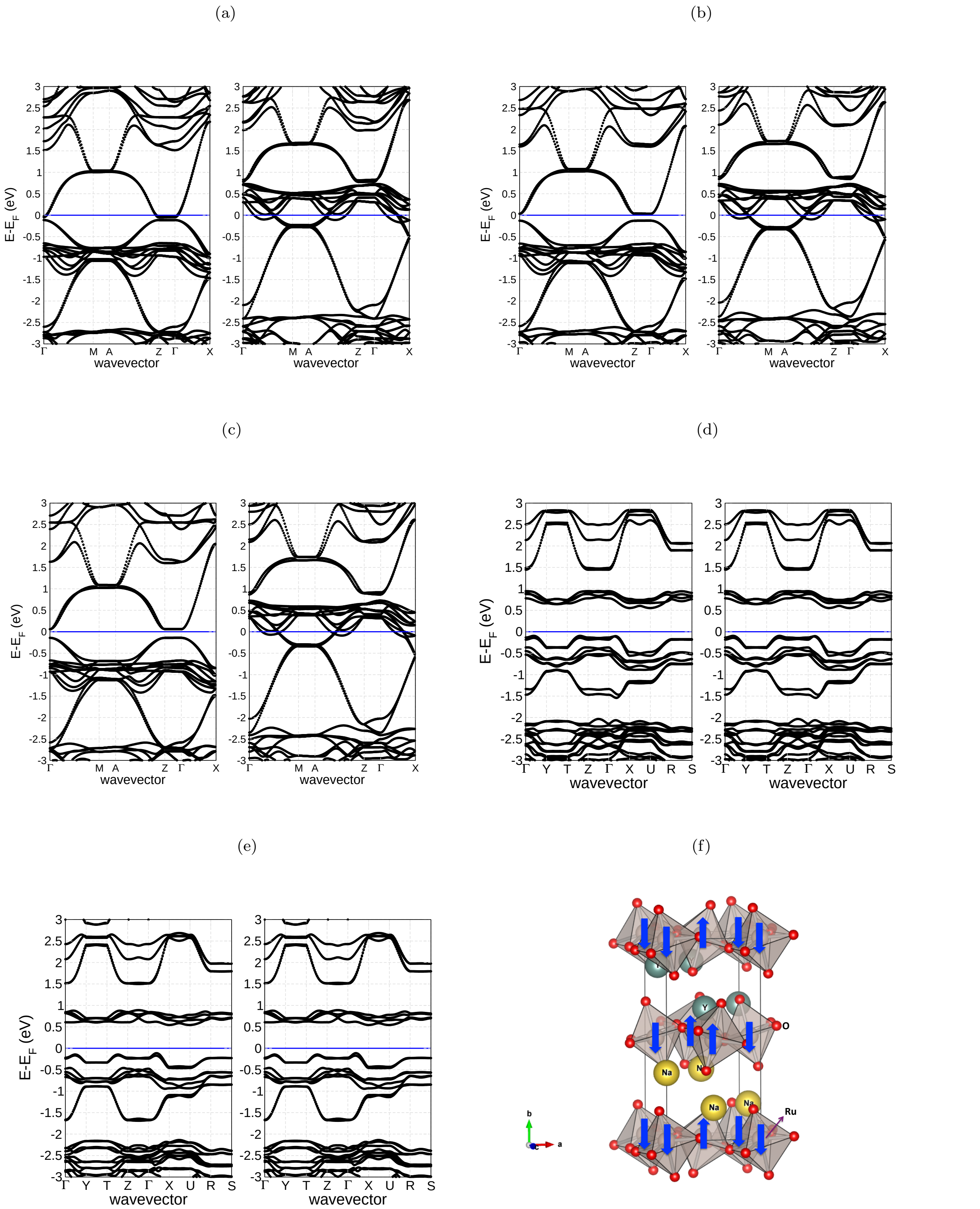

Supplementary Figure 11 | Electronic band structure data (spin-up channel in the left and spin-down channel in the right) for NaRRuO$_4$. (a) R=La (b) R=Pr and (c) R=Nd in $P\bar{4}2_1m$ crystal structure and ferromagnetic spin order on the Ru-atom. (d) R=Gd and (e) R=Y in $Pca2_1$ crystal structure and anti-ferromagnetic (AFM) spin order on the in-plane Ru-atoms. All DFT calculations were performed using PBEsol exchange-correlation functional. Fermi level at 0 eV is shown as Blue line. (f) A schematic showing the AFM spin configuration (blue arrows) imposed on the Ru-atom.

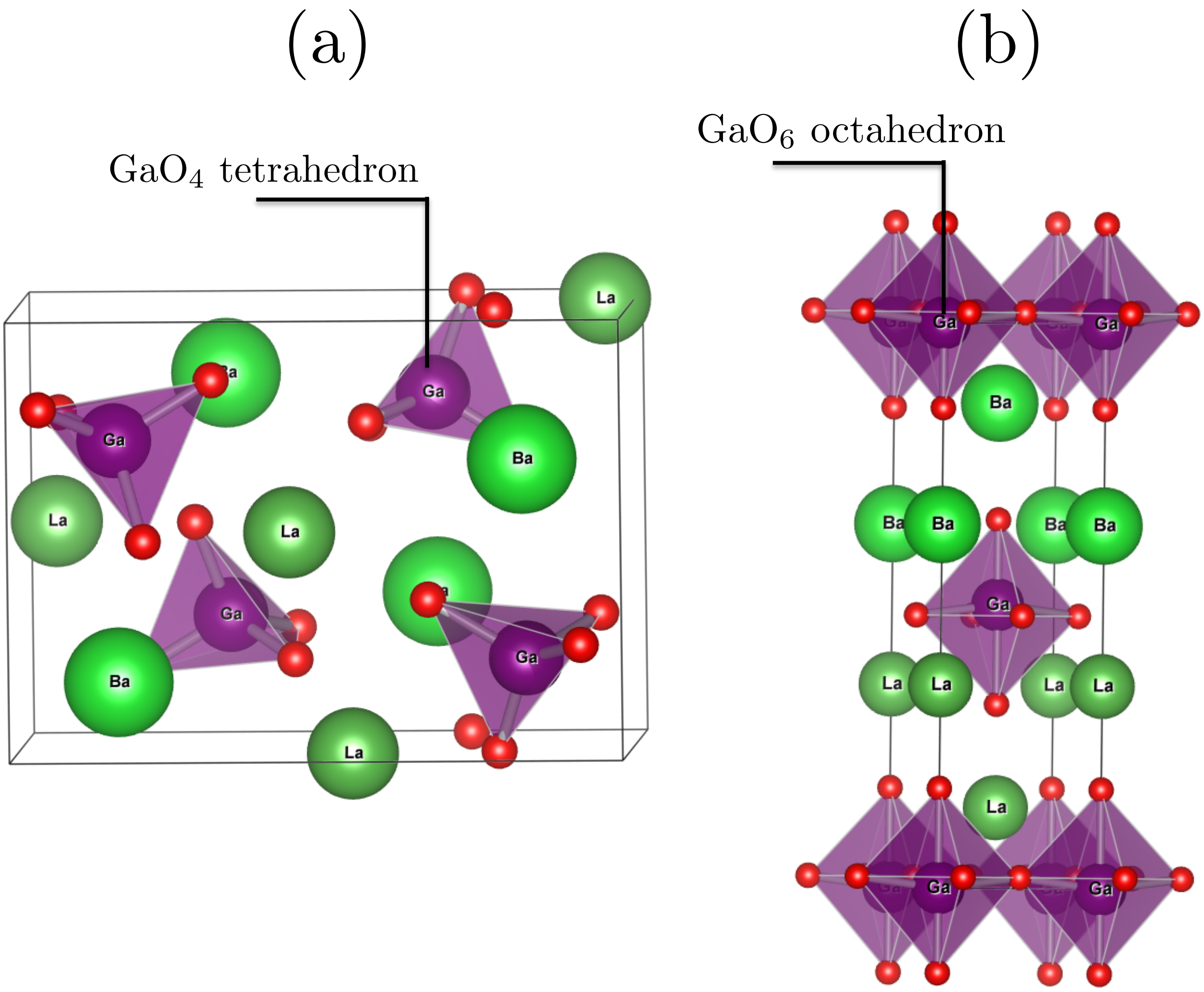

Supplementary Figure 12 | DFT optimized crystal structures for $BaLaGaO_4$ in two different atomic arrangements. (a) The oxygen atoms (red color) are in tetrahedral coordination with the central Ga atom and the GaO_4 tetrahedral units are isolated. The crystal structure belongs to the $P2_12_12_1$ space group. (b) The oxygen atoms (red color) are in octahedral coordination with the central Ga atom and the GaO_6 octahedral units are corner-connected in two-dimensions, which is typical of Ruddlesden-Popper (RP) structure-type. The RP crystal structure belongs to the $P4/nmm$ space group.

Supplementary Table 1 | Performance of five decision tree models on the training set and after 10-fold cross-validation (CV).

Decision tree model	Classification accuracy (in %)	
	Training set	10-fold CV
Bootstrap sample 1 (Tree 1, Supplementary Figure 4)	98.7	82.1
Bootstrap sample 2 (Tree 2, Supplementary Figure 5)	94.9	79.5
Bootstrap sample 3 (Tree 3, Supplementary Figure 6)	96.2	74.3
Bootstrap sample 4 (Tree 4, Supplementary Figure 7)	96.2	91.0
Bootstrap sample 5 (Tree 5, Supplementary Figure 8)	97.4	84.6

Supplementary Table 2 | IDs for A- and B-site elements in the Supplemental Excel Sheet. For e.g., IDs 1, 16, 22 correspond to $NaLaHfO_4$ chemical composition.

ID	A-site cation	ID	B-site cation
1	Na^{1+}	0	Fe^{2+}
2	K^{1+}	1	Co^{2+}
3	Rb^{1+}	2	Ni^{2+}
4	Cs^{1+}	3	Cu^{2+}
5	Tl^{1+}	4	Al^{3+}
6	Ag^{1+}	5	Sc^{3+}
7	Mg^{2+}	6	V^{3+}
8	Ca^{2+}	7	Cr^{3+}
9	Sr^{2+}	8	Mn^{3+}
10	Cd^{2+}	9	Fe^{3+}
11	Ba^{2+}	10	Co^{3+}
12	Hg^{2+}	11	Ni^{3+}
13	Pb^{2+}	12	Ti^{3+}
14	Y^{3+}	13	Ga^{3+}
15	Bi^{3+}	14	In^{3+}
16	La^{3+}	15	Ti^{4+}
17	Ce^{3+}	16	V^{4+}
18	Pr^{3+}	17	Cr^{4+}
19	Nd^{3+}	18	Mn^{4+}
20	Pm^{3+}	19	Zr^{4+}
21	Sm^{3+}	20	Ru^{4+}
22	Eu^{3+}	21	Sn^{4+}
23	Gd^{3+}	22	Hf^{4+}
24	Tb^{3+}	23	Ir^{4+}
25	Dy^{3+}	24	Nb^{5+}
26	Ho^{3+}	25	Ta^{5+}
27	Er^{3+}		
28	Tm^{3+}		
29	Yb^{3+}		
30	Lu^{3+}		

Supplementary Table 3 | Difference in total energy (ΔE, in meV/atom) with respect to the lowest energy structure. Space group with ΔE=0 is the ground state structure. CS and NCS stand for centrosymmetric and noncentrosymmetric structures, respectively. FM stands for ferromagnetic spin order, imposed on the Ir-atom. For $NaCaTaO_4$, the CS ground state $P\bar{4}$ becomes NCS $P\bar{4}2_1m$ when the tolerance for the lattice constants are set at 0.01 or lower.

n=1 RP	Space group	ΔE (meV/atom)	Description
$NaLaHfO_4$	$P4/nmm$	+9.62	High symmetry (CS)
	$Pmn2_1$	+7.07	NCS
	$P2_1/m$	+7.06	CS
	$P\bar{4}2m$	+9.37	NCS
	$P\bar{4}2_1m$	0	NCS
	$P2$	+0.001	NCS
	$Pca2_1$	+1.86	NCS
$NaLaZrO_4$	$P4/nmm$	+17.4	High symmetry (CS)
	$P\bar{4}2_1m$	0	NCS
	$P2_12_12$	0	NCS
	$Pbcm$	+13.21	CS
	$Pca2_1$	+1.35	NCS
$KLaIrO_4$ (FM)	$P4/nmm$	+0.04	High symmetry (CS)
	$P\bar{4}2_1m$	+0.03	NCS
	$Pbcm$	0	CS
	$Pca2_1$	+0.04	NCS
	$Ibca$	+0.90	NCS
$NaLaIrO_4$ (FM)	$P4/nmm$	+5.47	High symmetry (CS)
	$P\bar{4}2_1m$	0	NCS
	$Pbcm$	+0.78	CS
	$Pca2_1$	+0.28	NCS
$KBaNbO_4$	$P4/nmm$	+0.04	High symmetry (CS)
	$P\bar{4}2_1m$	+0.007	NCS
	$P2_1/m$	+0.04	CS
	$Pmn2_1$	+0.02	NCS
	$Pnma$	+0.03	CS
	Cc	+0.001	NCS
	$P2_1$	0	NCS
$SrYGaO_4$	$P4/nmm$	+27.81	High symmetry (CS)
	$Pmn2_1$	+26.86	NCS
	$P\bar{4}2_1m$	+0.01	NCS
	$P\bar{4}2m$	+24.17	NCS
	$I\bar{4}2m$	+3.71	NCS
	$Pnma$	+26.87	CS
	Pm	+6.78	NCS
	$P2_1$	0	NCS
$SrLaInO_4$	$P4/nmm$	+45.65	High symmetry (CS)
	$Pmn2_1$	+42.23	NCS
	$P2_1/m$	+45.38	CS
	$P2_1/c$	+43.98	CS
	$P\bar{4}2_1m$	0	NCS
	$P\bar{4}2m$	+30.88	NCS
	Pm	+35.2	NCS
	$Pca2_1$	+3.83	NCS
$NaCaTaO_4$	$P4/nmm$	+31.44	High symmetry (CS)
	$P1$	+29.11	NCS
	Pm	+25.15	NCS
	$P\bar{1}$	+30.55	CS
	$P\bar{4}$	+4.99	CS
	$P\bar{4}2m$	+10.14	NCS
	$Ibca$	+12.54	CS
	$Pcca$	+12.53	CS
	Pm	+28.74	NCS
	$Pca2_1$	0	NCS

SUPPLEMENTARY NOTE 1

We used PCA to reduce the dimensionality of the data from 22 to 8 column vectors, yet capturing $> 90\%$ of the variation in the data. Each PC is a linear combination of the weighed contribution of orbital radii, and we show all PC's in the Supplementary Figure 2. We now turn our attention to the decision tree shown in Supplementary Figure 7 and follow the path PC1 ≤ -2.6796 AND PC2 ≤ -0.1335 AND PC5 $\leq 0.152 \rightarrow \mathrm{X}_3^+(\eta_1, \eta_2)$ in the leaf node. From Supplementary Figure 2, the following orbitals are identified as important for predicting the irrep $\mathrm{X}_3^+(\eta_1, \eta_2)$ using our decision tree:

- PC1: Orbital radii A-5p, A-6s, A-4f, B-4s, B-3d, B-5s and B-4d are important, because their weighted contributions are relatively larger than that for other orbital radii.
- PC2: A-2p, A-3s, B-6s, B-5d, B-4p, B-5s and B-4d
- PC5: A-4p, A-5s, A-4d, A-5p, A-6s, B-4s, B-3d, B-5s and B-4d

Projected density of states (PDOS) from DFT calculations for RP compounds with $\mathrm{X}_3^+(\eta_1, \eta_2)$ octahedral distortions in the ground state would allow us to validate this finding. Exploring changes in orbital bandwidths and shifts in their center-of-mass would permit us to glean insights necessary for describing the stability of a crystal structure (or distortions). Thus, one can potentially extract physical meaning from PCA and decision trees. We do not carry out the electronic structure calculations here, because we anticipate the decision trees to evolve as more compounds are validated and fed back for re-training our models.

SUPPLEMENTARY NOTE 2

Confusion matrix data for the five decision trees based on 10-fold cross-validation. Rows represent observed or true irrep labels and columns indicate output from the decision tree classifier. Diagonal elements represent the number of compositions that show perfect agreement between the true label and the classifier output.

Decision Tree 1 (Supplementary Figure 4)

	P4	NO	X311	X2X3	X301	X312
P4	3	0	0	1	1	1
NO	0	23	2	0	4	0
X311	0	1	8	0	0	0
X2X3	0	0	0	11	0	0
X301	0	2	2	0	14	0
X312	0	0	0	0	0	5

Decision Tree 2 (Supplementary Figure 5)

	NO	X311	X301	X312	P4	X2X3
NO	26	2	2	1	0	1
X311	1	5	1	1	0	0
X301	2	0	14	0	0	0
X312	2	2	0	3	0	0
P4	0	1	0	0	4	0
X2X3	0	0	0	0	0	10

Decision Tree 3 (Supplementary Figure 6)

	X2X3	X311	X301	NO	P4	X312
X2X3	9	0	0	0	0	0
X311	0	4	2	0	0	0
X301	0	0	8	2	2	0
NO	0	0	5	28	0	3
P4	1	0	1	2	5	0
X312	0	0	0	2	0	4

Decision Tree 4 (Supplementary Figure 7)

	NO	X312	X301	X311	X2X3	P4
NO	29	2	1	1	0	0
X312	0	12	0	0	0	0
X301	0	0	15	0	0	0
X311	0	1	0	9	0	0
X2X3	0	0	0	0	6	0
P4	0	0	1	0	1	0

Decision Tree 5 (Supplementary Figure 8)

	NO	P4	X301	X311	X2X3	X312
NO	33	0	1	2	0	2
P4	0	2	1	0	1	0
X301	2	0	12	0	0	0
X311	1	0	0	7	0	0
X2X3	0	0	0	0	7	0
X312	0	0	1	1	0	5

SUPPLEMENTARY NOTE 3

The synthetic minority class oversampling (SMOTE) was performed using WEKA for the two irrep labels, P_4 and X_3^+ (η_1, η_2) using three nearest neighbors (k) and default random seed. Three and six synthetic data points were augmented for P_4 and X_3^+ (η_1, η_2) labels, respectively. The five bootstrapped samples for classification learning were generated using the function `sample()` in R. We used the `set.seed()` function in R and the following five arguments were passed: 877, 963, 837, 212 and 505, for generating the random samples. Default metaparameters were used for J48 decision tree induction.

All potential NCS chemical compositions predicted from the 5 decision trees are given separately in an Excel Sheet that can downloaded from figshare.[1] The chemical IDs in the Excel sheet should be cross-referenced with Supplementary Table 2 to identify the exact chemical composition. The starting dataset with 69 RP chemical compositions, 22 orbital radii features and the irrep label is also given in figshare.[1] Full dataset with 3,253 chemical compositions that include 69 original, 9 from SMOTE and 3,175 virtual compounds is given in the Excel sheet that can be downloaded from figshare.[1] The data for experimentally known RP compounds were collected and organized from surveying the literature.[2–26]

SUPPLEMENTARY NOTE 4

The decomposition reaction pathways for the RP compositions explored in this work from the Grand Canonical Linear Programming (GCLP) method as implemented in the Open Quantum Materials Database (OQMD).

Decomposition Reactions from OQMD:

$$\mathrm{CaO + CaIrO_3 \longrightarrow Ca_2IrO_4}$$

$$\begin{aligned}
\mathrm{0.25Na_4SnO_4 + 0.375La_2Sn_2O_7 + 0.125La_2O_3} &\longrightarrow \mathrm{NaLaSnO_4}\\
\mathrm{0.20Na_4SnO_4 + 0.40Pr_2Sn_2O_7 + 0.20NaPrO_2} &\longrightarrow \mathrm{NaPrSnO_4}\\
\mathrm{0.20Na_4SnO_4 + 0.40Nd_2Sn_2O_7 + 0.20NaNdO_2} &\longrightarrow \mathrm{NaNdSnO_4}\\
\mathrm{0.20Na_4SnO_4 + 0.40Gd_2Sn_2O_7 + 0.20NaGdO_2} &\longrightarrow \mathrm{NaGdSnO_4}\\
\mathrm{0.25Na_4SnO_4 + 0.375Y_2Sn_2O_7 + 0.125Y_2O_3} &\longrightarrow \mathrm{NaYSnO_4}
\end{aligned}$$

$$\begin{aligned}
\mathrm{0.125Ru + 0.25Na_2RuO_3 + 0.125RuO_2 + 0.50NaLa_2RuO_6} &\longrightarrow \mathrm{NaLaRuO_4}\\
\mathrm{0.05Ru + 0.50Na_2RuO_3 + 0.05RuO_2 + 0.20Pr_5Ru_2O_{12}} &\longrightarrow \mathrm{NaPrRuO_4}\\
\mathrm{0.125Ru + 0.25Na_2RuO_3 + 0.125RuO_2 + 0.50NaNd_2RuO_6} &\longrightarrow \mathrm{NaNdRuO_4}\\
\mathrm{0.50Na_2RuO_3 + 0.50RuO_2 + 0.50Gd_2O_3} &\longrightarrow \mathrm{NaGdRuO_4}\\
\mathrm{0.50Na_2RuO_3 + 0.50RuO_2 + 0.50Y_2O_3} &\longrightarrow \mathrm{NaYRuO_4}
\end{aligned}$$

$$\begin{aligned}
\mathrm{0.50La_2Hf_2O_7 + 0.50Na_2O} &\longrightarrow \mathrm{NaLaHfO_4}\\
\mathrm{0.50La_2Zr_2O_7 + 0.50Na_2O} &\longrightarrow \mathrm{NaLaZrO_4}\\
\mathrm{0.188Ir + 0.25NaIrO_3 + 0.0625Na_4IrO_4 + 0.50NaLa_2IrO_6} &\longrightarrow \mathrm{NaLaIrO_4}\\
\mathrm{0.75KIrO_3 + 0.0625K_4IrO_4 + 0.5La_2O_3 + 0.188Ir} &\longrightarrow \mathrm{KLaIrO_4}\\
\mathrm{0.333K_2O + 0.333Ba_3Nb_2O_8 + 0.333KNbO_3} &\longrightarrow \mathrm{KBaNbO_4}\\
\mathrm{0.25Ca_2Ta_2O_7 + 0.25NaTaO_3 + 0.25Na_3Ca_2TaO_6} &\longrightarrow \mathrm{NaCaTaO_4}\\
\mathrm{0.50SrO + 0.50La_2O_3 + 0.50SrIn_2O_4} &\longrightarrow \mathrm{SrLaInO_4}\\
\mathrm{0.50Y_2O_3 + 0.25Sr_3Ga_4O_9 + 0.25SrO} &\longrightarrow \mathrm{SrYGaO_4}\\
\mathrm{BaO + LaGaO_3} &\longrightarrow \mathrm{BaLaGaO_4}
\end{aligned}$$

For all NaRSnO$_4$, where R=La, Pr, Nd, Gd and Y, the ground state $P\bar{4}2_1m$ space group was considered to compute the total energies from DFT. For $NaLaRuO_4$, $NaPrRuO_4$ and $NaNdRuO_4$ we considered $P\bar{4}2_1m$ space group in the ferromagnetic spin order. On the other hand, for $NaGdRuO_4$ and $NaYRuO_4$ we considered $Pca2_1$ space group in the ferromagnetic spin order. In the case of Ca_2IrO_4 RP compound, we considered both the theoretical ground state ($Pbca$) and high-symmetry ($I4/mmm$) structures. For the remaining RP compounds, the ground state structures given in Supplementary Table 3 were used. We also note that the RP $BaLaGaO_4$ is +23.7 meV/atom above the convex hull relative to another compound with identical chemical formula, but different atomic arrangement (containing GaO_4 tetrahedral units and its crystal structure belongs to $P2_12_12_1$ space group, see Supplementary Figure 12). Thus, non-equilibrium synthesis techniques may be required to stabilize the RP phase in $BaLaGaO_4$.

In the main manuscript, we provide the decomposition energy (ΔE^D) data for $KBaNbO_4$ in Table 7. We calculated ΔE^D to be −832 meV/atom, which is too low (relative to other compounds in the same Table 7). To test the reliability of this data, we performed additional calculations using a different set of pseudopotentials (GBRV ultrasoft PBEsol pseudopotentials[27]), but we used the same crystal structures ($P2_1$, $Fm\bar{3}m$, $R\bar{3}$m and $P4mm$ for $KBaNbO_4$, K_2O, $Ba_3Nb_2O_8$ and $KNbO_3$, respectively) and decomposition reaction. We then fully relaxed the structures using the

GBRV pseudopotentials and recalculated the value for ΔE^D to be −826 meV/atom, which is similar to that reported in Table 7.

[1] P. V. Balachandran, J. Young, T. Lookman, and J. M. Rondinelli, (2016), 10.6084/m9.figshare.4264190.v1.
[2] J. Kafalas and J. Longo, Journal of Solid State Chemistry **4**, 55 (1972).
[3] J. Longo and P. Raccah, Journal of Solid State Chemistry **6**, 526 (1973).
[4] J. Gopalakrishnan, G. Colsmann, and B. Reuter, Journal of Solid State Chemistry **22**, 145 (1977).
[5] K. Singh, P. Ganguly, and J. Goodenough, Journal of Solid State Chemistry **52**, 254 (1984).
[6] M. Leonowicz, K. Poeppelmeier, and J. Longo, Journal of Solid State Chemistry **59**, 71 (1985).
[7] A. B. Austin, L. G. Carreiro, and J. V. Marzik, Materials Research Bulletin **24**, 639 (1989).
[8] J. D. Axe, A. H. Moudden, D. Hohlwein, D. E. Cox, K. M. Mohanty, A. R. Moodenbaugh, and Y. Xu, Phys. Rev. Lett. **62**, 2751 (1989).
[9] Y. Takeda, R. Kanno, M. Sakano, O. Yamamoto, M. Takano, Y. Bando, H. Akinaga, K. Takita, and J. Goodenough, Materials Research Bulletin **25**, 293 (1990).
[10] J.-C. Bouloux, J.-L. Soubeyroux, G. L. Flem, and P. Hagenguller, Journal of Solid State Chemistry **38**, 34 (1981).
[11] J. Rodriguez-Carvajal, M. T. Fernandez-Diaz, and J. L. Martinez, Journal of Physics: Condensed Matter **3**, 3215 (1991).
[12] A. Nozaki, H. Yoshikawa, T. Wada, H. Yamauchi, and S. Tanaka, Phys. Rev. B **43**, 181 (1991).
[13] M. K. Crawford, R. L. Harlow, E. M. McCarron, W. E. Farneth, J. D. Axe, H. Chou, and Q. Huang, Phys. Rev. B **44**, 7749 (1991).
[14] L. Walz and F. Lichtenberg, Acta Crystallographica Section C **49**, 1268 (1993).
[15] V. Voronin, A. Mirmelstein, V. Kozhevnikov, and B. Goshchitskii, Physica C: Superconductivity **218**, 407 (1993).
[16] M. Braden, G. André, S. Nakatsuji, and Y. Maeno, Phys. Rev. B **58**, 847 (1998).
[17] G. K. Rozenberg, A. P. Milner, M. P. Pasternak, G. R. Hearne, and R. D. Taylor, Phys. Rev. B **58**, 10283 (1998).
[18] K. Ramanujachary, J. S. IV, I. Fawcett, P. Shuk, M. Greenblatt, M. Croft, I. Nowik, R. Herber, and S. Khalid, Materials Research Bulletin **34**, 803 (1999).
[19] R. E. Schaak and T. E. Mallouk, Journal of Solid State Chemistry **161**, 225 (2001).
[20] O. Friedt, M. Braden, G. André, P. Adelmann, S. Nakatsuji, and Y. Maeno, Phys. Rev. B **63**, 174432 (2001).
[21] W. Fu, D. Visser, and D. IJdo, Journal of Solid State Chemistry **169**, 208 (2002).
[22] R. Patel, C. Simon, and M. T. Weller, Journal of Solid State Chemistry **180**, 349 (2007).
[23] M. C. Knapp, *Investigations into the structure and properties of ordered perovskites, layered perovskites, and defect pyrochlores*, Ph.D. thesis, The Ohio State University (2007).
[24] T. Qi, M. Ge, O. Korneta, S. Parkin, L. D. Long, and G. Cao, Journal of Solid State Chemistry **184**, 893 (2011).
[25] T. F. Qi, O. B. Korneta, S. Parkin, J. Hu, and G. Cao, Phys. Rev. B **85**, 165143 (2012).
[26] H. Akamatsu, K. Fujita, T. Kuge, A. Sen Gupta, A. Togo, S. Lei, F. Xue, G. Stone, J. M. Rondinelli, L.-Q. Chen, I. Tanaka, V. Gopalan, and K. Tanaka, Phys. Rev. Lett. **112**, 187602 (2014).
[27] K. F. Garrity, J. W. Bennett, K. M. Rabe, and D. Vanderbilt, Computational Materials Science **81**, 446 (2014).
